# Pirogoff amputation is a viable option to maintain ambulation in chronic limb-threatening ischemia with extensive midfoot tissue loss: a report of two cases

**DOI:** 10.1093/jscr/rjae180

**Published:** 2024-03-21

**Authors:** Masaya Sano, Hokuto Morii, Takashi Endo, Masaru Kimura, Satoshi Yamamoto, Takuya Hashimoto, Juno Deguchi

**Affiliations:** Department of Vascular Surgery, Saitama Medical Center, Saitama Medical University, 1981 Kamoda, Kawagoe, Saitama 350-0844, Japan; Department of Emergency and Critical Care Medicine, Saitama Medical Center, Saitama Medical University, 1981 Kamoda, Kawagoe, Saitama 350-0844, Japan; Department of Vascular Surgery, Saitama Medical Center, Saitama Medical University, 1981 Kamoda, Kawagoe, Saitama 350-0844, Japan; Department of Vascular Surgery, Saitama Medical Center, Saitama Medical University, 1981 Kamoda, Kawagoe, Saitama 350-0844, Japan; Department of Vascular Surgery, Saitama Medical Center, Saitama Medical University, 1981 Kamoda, Kawagoe, Saitama 350-0844, Japan; Department of Vascular Surgery, Saitama Medical Center, Saitama Medical University, 1981 Kamoda, Kawagoe, Saitama 350-0844, Japan; Department of Vascular Surgery, Saitama Medical Center, Saitama Medical University, 1981 Kamoda, Kawagoe, Saitama 350-0844, Japan

**Keywords:** Pirogoff amputation, CLTI, diabetic foot

## Abstract

Eliminating necrotic and infected tissues is crucial for limb salvage in patients with chronic limb-threatening ischemia (CLTI). However, extensive lesions that involve the midfoot frequently result in transtibial amputation, restricting ambulation and independent life. The Modified Pirogoff amputation, which includes a 90° rotation of the calcaneus and fixation with the tibia, has good functional outcomes in trauma cases. Here, we report two patients with CLTI successfully managed by a combination of revascularization and modified Pirogoff amputation, resulting in preserved ambulation without a prosthesis. Modified Pirogoff amputation may be a viable option in revascularized CLTI with extensive tissue loss of the midfoot.

## Introduction

Chronic limb-threatening ischemia (CLTI), which represents the most severe form of peripheral artery disease, is associated with high rates of limb loss [1]. Infection and ischemia are crucial factors contributing to subsequent above-ankle amputation for CLTI. Therefore, in addition to restoration of blood flow, aggressive removal of necrotic or infected tissues is required to achieve limb salvage in patients with extensive foot lesions.

On the other hand, functional efficacy of amputations proximal to the midfoot, i.e. Lisfranc and Chopart amputation, remain controversial [2], due to deformities and recurrent ulcers [3, 4]. Moreover, Syme ankle disarticulation has not been well accepted, mainly because of gait disturbance by heel pad migration and difficulty in walking due to discrepancies in limb length [5]. Therefore, most surgeons believe that hindfoot amputations are rarely indicated, resulting in a choice of below-the-knee transtibial amputation in patients with extensive foot lesions due to CLTI. However, after this major amputation, only 40% of patients maintain ambulation with the aid of a prostheses [6].

To minimize leg length discrepancy after amputation, in 1854, Nikolai Pirogoff first described a unique hindfoot amputation technique with 90° rotation of the calcaneus [7]. In the actual technique, the skin incision was made distal to the Chopart joint (on the plantar side, the skin is incised distal enough to create an adequate skin flap). Next, the talus is released and extracted. After the articular surface of the calcaneus is resected, the calcaneus is brought anteriorly under the tibia and rotated 90˚. Finally, a tibio-calcaneus arthrodesis is performed with three fixation nails. With modification of the bone fixation method [8], this technique has shown good functional outcomes with minimal leg length discrepancy for patients with trauma. However, there have been few reports of this method’s efficacy in patients with leg ischemia [9–12].

Here, we present two CLTI cases with extensive diabetic foot gangrene, who maintained ambulation by a combination of targeted revascularization to the posterior tibial artery and modified Pirogoff amputation.

## Case presentation

### Case 1

**Figure 1:**
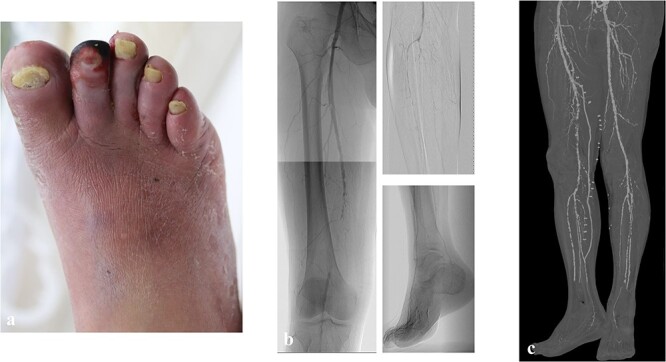
The preoperative clinical picture of case 1; (a) gangrene of the second digit with infection; (b) preoperative angiography; the GLASS stage was II (TAP: posterior tibial artery, FP2, IP2); (c) postoperative computed tomography; superficial femoral artery-posterior tibial artery bypass with a great saphenous vein was performed.

A 60-year-old man with diabetes mellitus and end-stage renal failure following 10-year maintenance hemodialysis was hospitalized for infected gangrene of the second digit. He had a history of percutaneous coronary intervention for ischemic heart disease. According to preoperative evaluation, his WIfI stage was 4 (wound grade 2, ischemia grade 3, foot infection grade 2), and the GLASS stage was II (target artery path: posterior tibial artery, femoropopliteal grade 2, infrapopliteal grade 2) (Fig. 1a and b) as assessed using the Global Vascular Guidelines [1]. Revascularization (superficial femoral artery-posterior tibial artery bypass with great saphenous vein) and open amputation of the second digit were performed (Fig. 1c).

**Figure 2:**
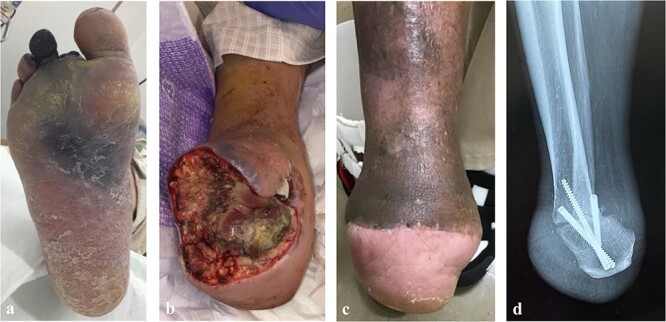
The postoperative clinical picture of case 1; (a) the infection spread to the third, fourth digits, and plantar side; (b) the clinical picture of after transection at the Chopart joint; (c) the clinical picture after Pirogoff amputation; (d) lateral radiography after Pirogoff amputation.

After surgery, remnant infection became exacerbated and spread to the third and fourth digits, and the plantar midfoot (Fig. 2a). Additional amputation and drainage were performed to control the infection, resulting in transection at the Chopart joint (Fig. 2b). Then, the modified Pirogoff technique was applied to achieve wound healing. After starting weight-free walking 1 month after surgery, it took 4 months for the wound to heal and 8 months for bone union (Fig. 2c and d). Ten months later, the patient could walk to the hospital with a cane and did not need a prosthesis at home.

### Case 2

A 70-year-old man on maintenance hemodialysis due to diabetes mellitus was hospitalized for infected gangrene of the second digit 3 months after vein graft occlusion of the below-knee popliteal artery to posterior tibial artery bypass. The patient had a history of coronary artery bypass grafting for ischemic heart disease. Open amputation was done immediately after admission, but the infection could not be controlled, and multiple additional debridements were required. Finally, there was a large tissue loss on the plantar side with partial forefoot amputation, leaving only the fourth and fifth digits.

Although angiography at admission revealed diffuse stenosis in the posterior tibial artery, run-off below the ankle arteries was maintained (GLASS stage II; target artery path: posterior tibial artery, femoropopliteal grade 0, infrapopliteal grade 3). After establishing an in-line flow to the ankle by balloon angioplasty in the posterior tibial artery, modified Pirogoff amputation was performed. Although one reintervention to the posterior tibial artery was required to facilitate wound healing, this patient also acquired short ambulation without a prosthesis at home.

## Discussion

Two CLTI cases with extended midfoot tissue loss were successfully managed by revascularization and by modified Pirogoff amputation. As a result of debridement to control infection, tissue loss became so significant that below-knee amputation was considered. After establishing in-line flow to the intact heel pads, modified Pirogoff amputation was performed. Both patients maintained short ambulation indoors without a prosthesis.

While there are other alternatives to hindfoot amputation, the advantage to Pirogoff amputation is that it provides an entire weight-bearing surface with minimal leg discrepancy compared with Syme amputation, thereby allowing short ambulation. In addition, tibio-calcaneal arthrodesis is performed in modified Pirogoff amputation, so there is no positional change of the heel pad due to shortening of the Achilles tendon, which leads to pressure ulcers as seen in other partial foot amputations (Lisfranc or Chopart) [13]. Even though peripheral neuropathy is a serious risk for pressure ulcers, in theory, Andronic *et al*. [9] reported that the risk of pressure ulcers in Pirogoff amputation did not increase even if patients had peripheral neuropathy.

Though modified Pirogoff amputation has been reported to achieve favorable long-term functional outcomes with trauma patients, there have been few reports involving CLTI patients [9, 10]. This is because attaining primary healing after amputation at any level is challenging in ischemic limbs [1], and we believe that good run-off of the posterior tibial artery is essential for Pirogoff amputation. Moreover, few CLTI patients are ambulatory and able to maintain activities of daily living (ADL) in the first place. Although both patients strongly requested to leave their leg as long as possible cases met the criteria for Pirogoff amputation, in that their heel pads and posterior tibial arteries were intact, both still needed a long time for the wound to heal and to achieve bone union [9]. In one of our cases, after removal of the fixation nail, the wound became ulcerated, and it took 14 months to heal after amputation. However, both cases walk outside with protheses, the two cases have attained short ambulation indoors without prostheses (e.g. enabling daily life indoors, such as walking to the toilet and moving around the room), for 16 and 13 months.

In both cases, the patients were relatively young and preserved preoperative ADL, and the diabetic gangrene factor was more significant than the ischemic factor. Their revascularized targets were the posterior tibial artery, and the heel pads were intact. Particularly in Japan, there is still a belief that it is a duty of filial piety not to harm the precious body given by parents, and understandably, many people wish to preserve their limbs as long as possible. It is true that Pirogoff amputation requires longer wound healing than below-knee amputation, but it allows for indoor walking without protheses. Pirogoff amputation may be a viable option in CLTI patients with extensive midfoot tissue loss, provided that inline blood flow to the intact heel pad is achievable.
