# Correction to: Pirogoff amputation is a viable option to maintain ambulation in chronic limb-threatening ischemia with extensive midfoot tissue loss: a report of two cases

**DOI:** 10.1093/jscr/rjae240

**Published:** 2024-07-13

This is a correction to: Masaya Sano, Hokuto Morii, Takashi Endo, Masaru Kimura, Satoshi Yamamoto, Takuya Hashimoto, Juno Deguchi, Pirogoff amputation is a viable option to maintain ambulation in chronic limb-threatening ischemia with extensive midfoot tissue loss: a report of two cases, *Journal of Surgical Case Reports*, Volume 2024, Issue 3, March 2024; https://doi.org/10.1093/jscr/rjae180

An earlier version of this article was published in error. The article has been updated online with the following changes:

In the Abstract, the third sentence should read: ``Modified Pirogoff amputation may be a viable option in revascularized CLTI with extensive tissue loss of the midfoot.'' instead of: ``Modified Pirogoff amputation may be a good alternative in revascularized CLTI with extensive tissue loss of the midfoot.''

In the third paragraph of the Introduction, four additional sentences have been added after the first sentence: ``In the actual technique, the skin incision was made distal to the Chopart joint (on the plantar side, the skin is incised distal enough to create an adequate skin flap). Next, the talus is released and extracted. After the articular surface of the calcaneus is resected, the calcaneus is brought anteriorly under the tibia and rotated 90°. Finally, a tibio-calcaneus arthrodesis is performed with three fixation nails.''

In the third paragraph of section Discussion, the fourth sentence should read: ``Although both patients strongly requested to leave their leg as long as possible both cases met the criteria for Pirogoff amputation, in that their heel pads and posterior tibial arteries were intact, both still needed a long time for the wound to heal and to achieve bone union [9].'' Instead of: ``Although both cases met the criteria for Pirogoff amputation, it still took a long time for the wound to heal and to achieve bone union [9].'' And the sixth sentence should read: ``However, both cases walk outside with prostheses, the two cases have attained short ambulation indoors without prostheses (e.g. enabling daily life indoors, such as walking to the toilet and moving around the room), for 16 and 13 months.'' instead of: ``However, two cases have attained short ambulation indoors without prostheses for 16 and 13 months.'' In the final paragraph, after the second sentence, two additional sentences have been added: ``Particularly in Japan, there is still a belief that it is a duty of filial piety not to harm the precious body given by parents, and understandably, many people wish to preserve their limbs as long as possible. It is true that Pirogoff amputation requires longer wound healing than below-knee amputation, but it allows for indoor walking without protheses.''

The emendations have been made to the article.

